# Appreciating animal induced pluripotent stem cells to shape plant cell reprogramming strategies

**DOI:** 10.1093/jxb/erae264

**Published:** 2024-06-13

**Authors:** Jana Wittmer, Renze Heidstra

**Affiliations:** Cell and Developmental Biology, cluster Plant Developmental Biology, Wageningen University & Research, Droevendaalsesteeg 1, 6708 PB, Wageningen, The Netherlands; Cell and Developmental Biology, cluster Plant Developmental Biology, Wageningen University & Research, Droevendaalsesteeg 1, 6708 PB, Wageningen, The Netherlands; CEFOBI-CONICET, Universidad Nacional de Rosario, Argentina

**Keywords:** *De novo* shoot organogenesis, developmental regulators, induced pluripotent stem cells (iPSCs), regeneration, somatic embryogenesis, stem cells

## Abstract

Animals and plants have developed resilience mechanisms to effectively endure and overcome physical damage and environmental challenges throughout their life span. To sustain their vitality, both animals and plants employ mechanisms to replenish damaged cells, either directly, involving the activity of adult stem cells, or indirectly, via dedifferentiation of somatic cells that are induced to revert to a stem cell state and subsequently redifferentiate. Stem cell research has been a rapidly advancing field in animal studies for many years, driven by its promising potential in human therapeutics, including tissue regeneration and drug development. A major breakthrough was the discovery of induced pluripotent stem cells (iPSCs), which are reprogrammed from somatic cells by expressing a limited set of transcription factors. This discovery enabled the generation of an unlimited supply of cells that can be differentiated into specific cell types and tissues. Equally, a keen interest in the connection between plant stem cells and regeneration has been developed in the last decade, driven by the demand to enhance plant traits such as yield, resistance to pathogens, and the opportunities provided by CRISPR/Cas-mediated gene editing. Here we discuss how knowledge of stem cell biology benefits regeneration technology, and we speculate on the creation of a universal genotype-independent iPSC system for plants to overcome regenerative recalcitrance.

## Teaser

Here we discuss how knowledge of stem cell biology benefits the improvement of plant regeneration technology.

## Introduction

### The trouble with plant regeneration

Plants display continuous post-embryonic growth and development due to maintenance of stem cell pools in the apical meristems. *De novo* stem cell pools maintain the meristems of lateral roots and shoots, supporting the formation of a branched system of organs to ensure life-long development (Greb and Lohmann, 2016). The formation of *de novo* stem cell pools may also be induced upon wounding, and thereby allow the plant to redirect growth. Thus, wound repair can involve the sealing of wounds and the reconnection of vascular tissue, but also the establishment of a *de novo* meristem from somatic tissue at the wound site, giving rise to new organs or even entirely new individuals (Sinnott, 1960).

Historically, the regenerative capabilities of plants are harnessed for clonal propagation and genetic engineering to maintain and enhance crop traits. Clonal propagation allows the reproduction of plants while maintaining their identical genotype and thereby phenotypic properties. In nature this can occur through apomixis, defined as the asexual development of seeds that bypass meiosis and fertilization (Wang and Underwood, 2023) or by the regeneration of new plants (ramets) from vegetative parts such as roots, stolons, stems, rhizomes, or tubers (Silvertown, 2008). Artificial vegetative reproduction exploits these properties to maintain and multiply plants involving methods such as grafting, cuttings, and tissue culture (Bhojwani and Razdan, 1996). Genetic engineering in plants is generally initiated by introducing (foreign) DNA into single cells that may originate from various tissues such as leaves, cotyledons, roots, hypocotyls, pollen tube, apical meristems, and immature embryos. These pieces of tissue or organ are collectively called explants. Subsequently, the transformed cells are rendered regeneration competent, which denotes two major pathways to regenerate transformed cells into fully grown plants. The first one is via *de novo* shoot organogenesis (DNSO) which most often involves an intermediate callus stage that organizes itself to form a *de novo* shoot meristem from induced pluripotent cells, from which ultimately a plantlet can arise. The second pathway is via somatic embryogenesis that involves the acquisition of totipotency by a cell, leading to the formation of an embryo-like structure with both a root and shoot apical meristem, which may develop into a complete plantlet (Raghavan, 2004).

Despite advances in plant tissue culture and regeneration techniques, significant hurdles persist in clonal propagation and plant genetic engineering. Many plant species exhibit recalcitrance to established procedures, stemming from tissue-, species-, and genotype-specific variations in regenerative capabilities, a property not readily resolved given these multiple variables. For example, wild tomato species were found to regenerate faster compared with cultivated tomatoes (Lech *et al.*, 1996), and only after introgressing loci from the wild relative was enhanced regeneration observed in *Solanum pennellii* cv. Micro-Tom (Pinto *et al.*, 2017). In addition, the age of the explant emerges as a critical factor, which is particularly pronounced in monocots where regenerative cells are scarce and often found to be confined to the basal regions of immature leaf blades post-embryonically. Consequently, reliance on immature embryos for regeneration exacerbates the challenge, necessitating manual dissection from seeds. Furthermore, *in vitro* culture conditions can induce somaclonal variation, heritable (epi)genetic changes observed in regenerated plantlets, triggered by various stresses such as hormone imbalance, wounding, prolonged subculturing, and oxidative stress (Bairu *et al.*, 2011).Such variation jeopardizes genetic fidelity in crops, undermining the reliability of tissue culture-based approaches. For example, a study in rice showed that plants regenerated from mature embryos had significantly more sequence variation than those originating from zygotes and immature embryos, highlighting the choice of explant origin as one of the factors to reduce somaclonal variation (Ichikawa *et al.*, 2023). Furthermore, tissue cultures that are optimized for rapid growth may yield physiologically abnormal regenerants possibly reflecting epigenetic changes and therefore ill-suited for transfer to greenhouse or field conditions (Hazarika, 2006).

Together, these regeneration hurdles and their time-consuming and often species-specifc mitigation highlight the necessity of innovative strategies to unlock the full potential of *in vitro* plant cell reprogramming. Here we may benefit from the concepts and progress in animal regeneration studies and take stem cell properties as a starting point to reshape regeneration strategies.

## Returning mature cells to the stem cell state

The discovery that toti- or pluripotency can be induced originated from the question of whether all cell types possessed the same set of genes. The definite experiment at the time was to test whether a somatic nucleus could functionally replace the zygote nucleus. By performing somatic cell nuclear transfer experiments in *Xenopus*, it was observed that the transplanted somatic nucleus was indeed able to lead to normal tadpole development, showing that reprogramming had occurred (Gurdon, 1962; Gurdon *et al.*, 1975). The outcomes of these experiments led to the general acknowledgement that development imposes reversible, rather than irreversible, epigenetic changes during cellular differentiation. However, the factors that could induce reprogramming and confer totipotency or pluripotency to somatic cells were yet to be discovered.

Pluripotency is a property of embryonic stem cells, that are derived from the inner cell mass of the mammalian blastocyst embryo. They have the capacity for unlimited self-renewal and the ability to differentiate into cells of all three germ layers (Evans and Kaufman, 1981; Martin, 1981). The initial cultivation of embryonic stem cells isolated from the blastocyst inner cell mass of mouse, and later human, depended on fibroblast feeder layers serving as a source for required growth factors, as cultivation with serum alone proved insufficient for the retrieval and maintenance of embryonic cells (Evans and Kaufman, 1981; Martin, 1981; Thomson *et al.*, 1998). Growth factors typically represent small proteins or peptides that regulate a diversity of processes during development, including proliferation, differentiation, survival, and cell migration (Gospodarowicz *et al.*, 1987; Kishimoto *et al.*, 1994; Stewart and Rotwein, 1996), and their use for differentiated cell cultures had already been shown to be essential. The successful culturing of embryonic stem cells paved the way for the molecular and biochemical examination of pluripotency (Lenardo *et al.*, 1989; Schöler *et al.*, 1989; Okamoto *et al.*, 1990).

An essential finding was the observation that certain transcription factors are responsible for lineage-specific development (Davis *et al.*, 1987). These transcription factors establish the expression of cell type-specific genes and at the same time repress lineage-inappropriate genes. The ectopic expression of these developmental regulators enabled the switch of one cell fate into another independent of their lineage or germ layer origin; for example, the conversion of a fibroblast cell with mesodermal origin into a neuronal cell normally derived from the ectoderm (Vierbuchen *et al.*, 2010). These transdifferentiation experiments set the intellectual framework for a systematic approach to find transcription factors that can revert a somatic cell back to a pluripotent cell state.

**Fig. 1.**
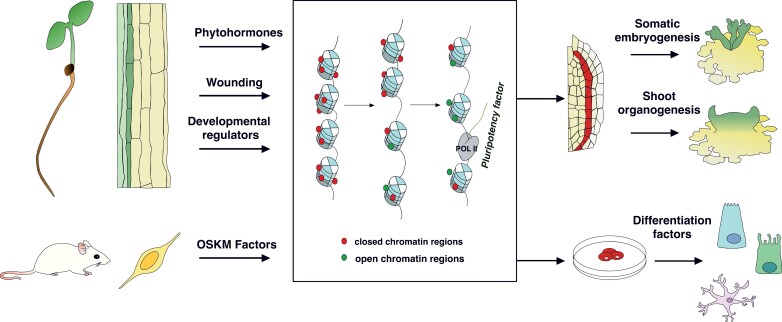
Framework of induced pluripotent stem cell (iPSC) generation in plants and animals. Somatic cells can be reprogrammed by exogenous hormones, wounding, and/or the application of developmental regulators. In mammals, different somatic cell strains can be reprogrammed to iPSCs through the combination of OSKM transcription factors. Both in animals and in plants a change in chromatin environment, typically including improving accessibility to pluripotency factors, is a prerequisite for iPSC (cells highlighted in red). These cells can then be reprogrammed to differentiate into various tissues and organs.

To find transcription factors able to induce pluripotency in adult cells, Yamanaka and Takahashi performed a systematic screen using a pool of 24 pluripotency-associated transcription factors to generate induced pluripotent stem cells (iPSCs) from mouse fibroblasts (Takahashi and Yamanaka, 2006). Successive elimination of individual transcription factors resulted in a minimal combination of four core regulators of pluripotency, namely octamer-binding protein (Oct4), SRY-box2 (Sox2), Krüppel-like factor (KLF4), and c-MYC, that are themselves highly expressed in embryonic stem cells (Takahashi and Yamanaka, 2006), and are collectively known as OSKM factors (Fig. 1).

Among the OSKM factors, the homeodomain factor OCT4 emerges as particularly crucial for preserving pluripotency *in vitro* and *in vivo*, with mutations in Oct4 causing the loss of pluripotency in the inner cell mass (Rodriguez *et al.*, 2007). Oct4 and Sox2 form a heterodimer to synergistically activate enhancers of other pluripotent stem cell-specific key players (Masui *et al.*, 2007). Remarkably, the role of Oct4 is evolutionarily highly conserved in the animal kingdom; human and axolotl Oct4 can replace mouse Oct4 in reprogramming, and human or mouse Oct4 combined with other OSKM genes can generate partially reprogrammed pluripotent stem cells in birds, zebrafish, and flies (Morrison and Brickman, 2006; Rosselló *et al.*, 2013). Furthermore, iPSCs have also been derived from different somatic cell types (Maherali *et al.*, 2007; Aasen *et al.*, 2008; Aoi *et al.*, 2008; Eminli *et al.*, 2008, 2009; Hanna *et al.*, 2008; Kim *et al.*, 2008; Stadtfeld *et al.*, 2008; Utikal *et al.*, 2009). This demonstrates not only that the fundamental features of the transcriptional networks governing pluripotency are evolutionarily conserved, but also that the induction of pluripotency seems universally applicable. Since the discovery of iPSCs, research in stem cell biology has quickly created new opportunities in disease modeling, drug discovery, and regenerative medicine (Fatima *et al.*, 2024). Pluripotent stem cells can even generate a complete embryo, but only when injected into or aggregated with an early embryo, thereby placing them in appropriate surroundings (Nagy *et al.*, 1993). However, only recently it was shown that human naive stem cells can self-organize to form embryo-like structures with characteristics of real 2-week-old embryos *in vitro* (Oldak *et al.*, 2023; Pedroza *et al.*, 2023; Weatherbee *et al.*, 2023). Apparently, these naive stem cells can bypass the earliest stages of embryo development that are typically observed upon fertilization, to directly enter post-implantation development. In the context of this review, these studies highlight the potential of *in vitro* stem cells as the single cell type origin of regeneration.

In plants, reprogramming of cell fate towards pluri- or totipotency is generally achieved by the exposure of explants to proper concentrations and ratios of the phytohormones auxin and cytokinin in tissue culture (Thorpe, 2007). Phytohormones are conceptually comparable with animal hormones, not resembling growth factors, and consist of relatively simple organic molecules. Of these, auxin and cytokinin are typically involved in plant developmental processes such as embryogenesis, meristem patterning, and stem cell maintenance, where they regulate gene expression through different signal transduction mechanisms and serve as pivotal signaling molecules (Jiménez, 2005; Su *et al.*, 2011; Miransari and Smith, 2014; Pacifici *et al.*, 2015). Their application in plant regeneration can lead to reprogramming of cells of different origin to adopt root or shoot stem cell-like fate and subsequently enter organogenesis. Such reprogramming is evident in a subset of tissue culture cells, by their expression of transcription factors known for their role in stem cell specification and maintenance in root or shoot stem cell niches (Box 1) (Sugimoto *et al.*, 2010, 2011; Rosspopoff *et al.*, 2017; Bustillo-Avendaño *et al.*, 2018; Gordon-Kamm *et al.*, 2019; Ikeuchi *et al.*, 2019; Zhai and Xu, 2021; Maren *et al.*, 2022; Ince and Sugimoto, 2023; Yan *et al.*, 2023). Specifically, the reprogramming process peaks in the formation of quiescence center (QC)-like cells that act as induced pluripotent stem cells from which new identities develop in subsequent regeneration events (Zhai and Xu, 2021; Yin *et al.*, 2024). In analogy to the mammalian iPSC studies, this raises the question of which, or which combination, of these developmental regulators are sufficient to confer and maintain stem cell fate *in vitro*, and how these genes can be instructive in subsequent regeneration of tissues and organs, or even embryos, to eventually develop a plant.

Box 1. **Plant stem cells and their spatial context in plant apical meristems**

*In planta*, stem cells reside in niches, microenvironments for their maintenance, and are located within meristems. The apical meristems are laid down during embryogenesis where hormonal signals converge with transcription factor regulation and ligand receptor signaling to specify and maintain the stem cell niche.

**The root stem cell niche**

The Arabidopsis root stem cell niche specification converges from two pathways involving auxin signaling through AUXIN RESPONSE FACTOR5/MONOPTEROS (ARF5/MP) and ARF7 (NON-PHOTOTROPIC HYPOCOTYL4) to mediate *ANTIGUMENTA-LIKE/PLETHORA* (*AIL/PLT*) expression that overlaps with that of the radial patterning genes *SHORTROOT/SCARECROW* (*SHR/SCR*) in the organizer. Root *PLT* genes are expressed in a gradient, peaking in the stem cell niche, and act in a dose-dependent manner, and the GROWTH-REGULATING FACTOR–GRF-INTERACTING FACTOR (GRF–GIF) repressive module may control *PLT* levels to sustain proliferation of meristem cells. In the organizer, complexes of PLT and SHR/SCR together with additional binding partners regulate expression of *WOX5* that is required for maintenance of surrounding stem cells. WOX5 acts in part by recruiting the co-repressor TOPLESS and HISTONE DEACETYLASE19 to repress the differentiation gene *CYCLIN DOF FACTOR* (*CDF4*). In addition, WOX5 action is counteracted by differentiation inducing ligand–receptor signaling (Shimotohno *et al.*, 2018; Shimotohno and Scheres, 2019; Pardal and Heidstra, 2021; Verma *et al.*, 2022).

**The shoot stem cell niche**

In Arabidopsis it was found that the WUSCHEL (WUS) transcription factor paralog WUSCHEL-RELATED HOMEOBOX 2 (WOX2) and its partially redundant family members specify a central domain as the future shoot stem cell niche by activating *HD-ZIPIII*-encoded transcription factors and enhancing cytokinin activity (Zhang *et al.*, 2017). HD-ZIPIII proteins are key determinants of shoot identity, and their spatial expression pattern is tightly controlled by multiple factors including miR165/166 family members (Wenkel *et al.*, 2007; Liu *et al.*, 2009; Smith and Long, 2010; Zhu *et al.*, 2011; Miyashima *et al.*, 2013; Beyene *et al.*, 2022). Subsequently, WUS becomes expressed in the organizer that moves into the stem cells to repress differentiation and activates *CLAVATA3-* (*CLV3*) encoded peptide expression. In turn, CLV3 ligand–receptor signaling delimits *WUS* expression in the organizer, together forming a central regulatory loop to maintain stem cell homeostasis (Greb and Lohman, 2016)*. SHOOT MERISTEMLESS* (*STM*) is broadly expressed in the meristem and acts at least in part via *ISOPENTENYL TRANSFERASE* (*IPT*) cytokinin biosynthesis gene expression and is required together with WUS to maintain the meristem. DORNROSCHEN/ENHANCER OF SHOOT REGENERATION1 (DRN/ESR) acts together with HD-ZIPIII to up-regulate *STM* in axillary meristem formation. Cytokinin acts via its response regulators *ARABIDOPSIS RESPONSE REGULATOR* (*ARR*) genes and positively regulates *WUS* expression. From the outer layer in the meristem, miR394 moves inward to target *LEAF CURLING RESPONSIVENE*SS (*LCR*), thereby conferring cellular competence to respond to WUS (Eshed Williams, 2021; Verma *et al.*, 2022; Hong and Fletcher, 2023).

**Figure:**
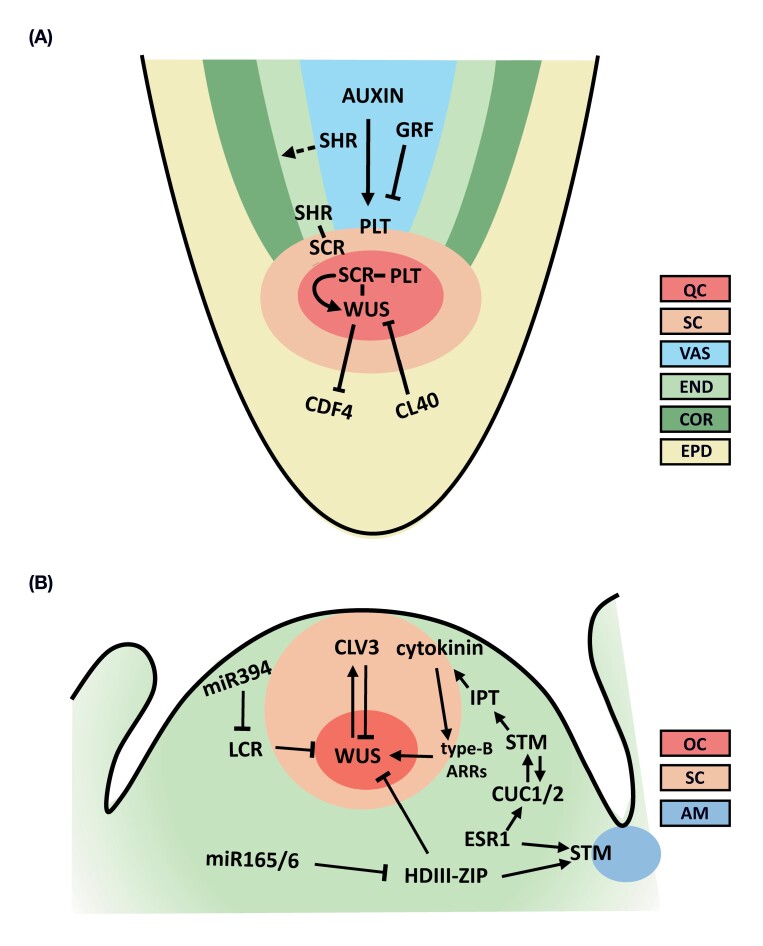
Schematic spatial overview of root and shoot stem cell niche organization. (A) Transcriptional networks mediating stem cell specification and maintenance in the root apical meristem. QC, quiescent center; SC, stem cells; VAS, vasculature; END, endodermis; COR, cortex; EPD, epidermis. (B) Gene networks involved in the formation and maintenance of the shoot apical meristem. OC, organizing center; AM, auxiliary meristem. Arrows and bar-head arrows represent activation and repression, respectively. A dashed arrow implicates protein movement.

## Three main classes of plant regeneration

The developmental regulators that are induced during reprogramming towards regeneration hold significant potential for their utilization in the induction of *de novo* meristem formation and somatic embryogenesis. However, it is crucial to comprehend the molecular mechanisms in which these genes are engaged for their efficient utilization. In this respect, regeneration can subjectively be divided into three forms of increasing complexity, with tissue repair upon damage being the most basic, followed by root and shoot organogenesis, and culminating in the reprogramming towards somatic embryogenesis and the direct outgrowth of a complete seedling.

### Wounding and its role in regeneration

Wounding is often the initial cue towards regeneration and can induce multiple response pathways in parallel that may correlate to the site or tissue that is damaged. For tissue repair after damage and organ loss, the activation of these wound-induced responses can ultimately lead to cell fate reprogramming and the restoration of appropriate cell types at the wounding site.

The plant hormone jasmonic acid (JA) is rapidly produced at the wound site and represents one of the signaling pathways to initiate repair processes. For example, after root tip excision or laser ablation of the QC, JA promotes the regeneration of a *de novo* stem cell niche (Zhou *et al.*, 2019). Specifically, JA induces ETHYLENE RESPONSE FACTORS (ERFs) ERF109 and ERF115 that together with a newly established auxin maxima promote regeneration by activating cell divisions and repatterning through the (up-)regulation of root stem cell genes (Zhou *et al.*, 2019).

**Fig. 2.**
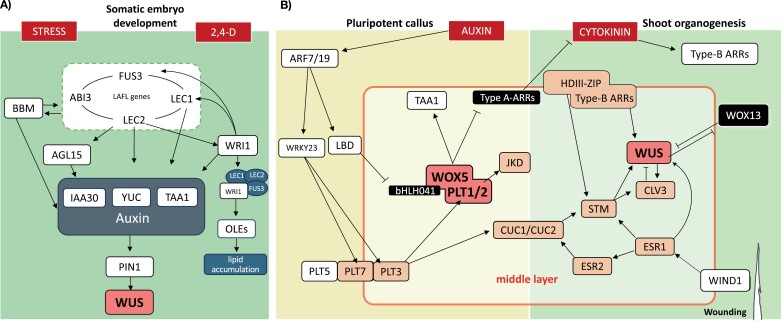
Molecular networks involved in hormone-induced regeneration.(A) Gene networks driving somatic embryogenesis can be initiated via auxin (2,4-D) and stress application. Several regulatory feedbacks between developmental regulators and the seed maturation program result in accumulation of auxin and lipid biosynthesis to maintain embryonic identity. Subsequent formation of totipotent cells requires WUS to allow embryo development. (B) *De novo* shoot organogenesis (DNSO) involves pluripotency acquisition in the callus middle layer on auxin-rich callus-inducing medium through the activation of root stem cell niche-related gene expression. Subsequent culture in cytokinin-rich conditions accomplishes the reprogramming into a shoot stem cell niche-related gene expression profile and the development of a shoot meristem followed by outgrowth. The color scheme relates to the schematic diagram in Box 1 and visually emphasizes the similarities in gene regulatory networks. Genes highlighted in black are negative regulators of DNSO. Arrows and bar-head arrows represent activation and repression, respectively.

Wound stress, for example induced upon cutting of the hypocotyl, can also promote callus formation and subsequently organogenesis. This represents a more complex form of regeneration compared with tissue repair, as new developmental programs need to be ectopically activated and organ structures are *de novo* reconstructed from naive cells without prior tissue context. The dedifferentiation factor gene *WOUND INDUCED DEDIFFERENTIATION1* (*WIND1*) was found to orchestrate wound-induced cellular reprogramming and callus proliferation (Fig. 2B) (Iwase *et al.*, 2015). Cytokinin accumulates prior to callus induction via increased transcription of its biosynthesis genes encoding enzymes such as IPT3 (Ikeuchi *et al.*, 2017). WIND- and cytokinin-induced molecular pathways converge to activate type-B Arabdidopsis response regulator (ARR)-mediated signaling to trigger the transcription of *CYCLIN-D3;1*, leading to cell cycle activation and the formation of callus tissue. WIND1 is probably activated by ERF115 which itself is up-regulated shortly after wounding and also required for regeneration from wound-induced callus (Heyman *et al.*, 2016; Ikeuchi *et al.*, 2017). At the same time PLT3, PLT5, and PLT7 are up-regulated to promote callus formation at the wound site, but their function seems to be independent of the WIND1 pathway (Ikeuchi *et al.*, 2017).Subsequently, shoot formation is promoted by WIND1 and cytokinin signaling through the activation of the transcription factor-encoding gene *DORNROSCHEN/ENHANCER OF SHOOT REGENERATION1* (*DRN/ESR1*) which in turn is involved in the induction of *(WUS*) (Fig. 2B) (Iwase *et al.*, 2017).

### Molecular insights from *de novo* shoot organogenesis

Phytohormone-induced regeneration via *de novo* shoot organogenesis is the most widely applied technique for biotechnological breeding purposes, particularly in dicotyledons. In contrast to wound-induced regeneration, *de novo* shoot organogenesis involves extensive auxin signaling and the activation of the lateral root initiation program. During phytohormone-induced regeneration, a portion of callus cells is induced to become pluripotent, followed by the formation of shoots or roots, depending on the ratio of exogenous hormone application. Pluripotency induction occurs during explant culture on auxin-rich callus-inducing medium (CIM) with the activation of root stem cell regulators (Atta *et al.*, 2009; Sugimoto *et al.*, 2011). With the transition to cytokinin-rich shoot-inducing medium (SIM), the expression of shoot-promoting factors is stimulated, leading to formation of shoot progenitors that can develop into shoots (Fig. 1). Finally, these shoots may be transferred to root-inducing medium (RIM) to initiate root regeneration, thereby eventually regenerating a whole plant body (Skoog and Miller, 1957; Feldmann and Marks, 1986).

In Arabidopsis, the induced callus on explants is formed from pericycle-like cells of aerial explants or from xylem pole pericycle cells of roots (Atta *et al.*, 2009; Sugimoto *et al.*, 2010). However, it should be noted that cell types of different origin and identity are able to gain regenerative competence, for example as observed in the regeneration from epidermis or mesophyll protoplasts (reviewed in Morinaka *et al.*, 2023). Callus formation is promoted by exposure to high auxin levels (Fig. 2B). As a result, bound AUXIN RESPONSE FACTOR (ARF) 7 and ARF19 are released to directly activate *LATERAL ORGAN BOUNDARIES DOMAIN* (*LBD*) gene expression and indirectly induce the transcriptional activator-encoding *WRKY23* (Fukaki *et al.*, 2005; Okushima *et al.*, 2007; Xu *et al.*, 2023). The LBD transcription factors induce the removal of the transcriptional repressor BASIC HELIX–LOOP–HELIX 041 (bHLH041), thereby derepressing the transcription of *PLT1/2* and *WOX5*. At the same time, WRKY23 directly up-regulates the expression of *PLT3/7* which, together with *PLT5*, promotes the expression of *PLT1/2* (Kareem *et al.*, 2015; Xu *et al.*, 2023). Instead of generating a mass of identical cells, the arising callus is organized into three distinct layers. It is in the middle cell layer where elevated levels of stem cell factors, that include PLT1/2 and WOX5, establish pluripotency, reminiscent of their role in the root stem cell niche (Fig. 2B; Box 1) (Zhai and Xu, 2021; Zhai *et al.*, 2023). Additionally, there PLT1/2 and WOX5 may act synergistically to promote endogenous auxin biosynthesis via the activation of *TRYPTOPHAN AMINOTRANSFERASE OF ARABIDOPSIS1* (*TAA1*), which could further enhance the maintenance of pluripotency (Zhai and Xu, 2021). Concomitantly, the sensitivity to cytokinin increases through the WOX5-mediated repression of type-A *ARR* genes, negative regulators of cytokinin signaling (Fig. 2B) (To *et al.*, 2004; Zhai and Xu, 2021). PLT3/5/7 further regulate the activation of the *CUPSHAPED COTYLEDON* (*CUC*) genes which are later involved in the activation of the shoot meristem (Fig. 2B) (Kareem *et al.*, 2015).

Upon transfer of the callus to SIM, the WOX5-mediated enhanced cytokinin sensitivity results in the up-regulation of *WUS*, again mainly in the callus middle cell layer (Fig. 2B) (Zhai and Xu, 2021). The B-type ARRs are the primary cytokinin response factors that directly activate *WUS* expression (Argyros *et al.*, 2008; Dai *et al.*, 2017; Zhang *et al.*, 2017). *WUS* expression during shoot formation from callus is limited to a few cells, despite the high cytokinin levels throughout the whole callus (Zhang *et al.*, 2017), indicating that additional factors are important to focus shoot stem cell niche establishment. One of these are the HD-ZIPIII transcription factors that complex with B-ARR proteins to spatially confine *WUS* expression within the callus (Zhang *et al.*, 2017; Yang *et al.*, 2022) reminiscent of their interaction in the shoot meristem (Fig. 2B; Box 1). *HD-ZIPIII* activation in the callus seems to be dependent on local auxin distribution by unknown mechanisms (Yang *et al.*, 2022). An antagonistically acting factor is the homeobox protein WOX13, that was found to actively repress *WUS*, while promoting cell differentiation via the activation of cell wall modifiers. Accordingly, knocking out *WOX13* enhanced the shoot regeneration capacity of *WUS* (Ogura *et al.*, 2023). WUS in turn represses *WOX13*, and this reciprocal inhibition leads to the formation of microterritories of fate specification (Fig. 2B) (Ogura *et al.*, 2023) The combination of positive and negative feedback loops on *WUS* thus focuses shoot meristem regeneration from the initially naive pluripotent stem cells in the callus middle layer.

### Molecular insights from somatic embryogenesis

Somatic embryogenesis involves *de novo* establishment of a bipolar embryo axis, ideally with the formation of a root pole and cotyledons (Horstman *et al.*, 2017a). Therefore, it arbitrarily presents the most complex form of regeneration, as it involves the direct re-initiation of a complete organism from somatic cells that are reprogrammed towards totipotency in the absence of fertilization.

The formation of somatic embryos can be induced *in vitro* by a variety of culture conditions including plant hormone treatments and/or stress conditions (Ikeda-Iwai *et al.*, 2003; Fehér, 2015). The most applied inducer is the synthetic auxin derivative 2,4-D, which also serves as a stress-inducing herbicide (Serbent *et al.*, 2019), and whose application results in an extensive reprogramming of the somatic cell transcriptome (Wang *et al.*, 2020). One of the differentially expressed transcription factor genes upon somatic embryogenesis initiation is *BABY BOOM* (*BBM*) (Boutilier *et al.*, 2002). *BBM* belongs to the *AIL/PLT* family of transcription factor genes and acts redundantly with *PLT2* during zygotic embryogenesis to define primordial cells that are precursors to all embryo cell types (Galinha *et al.*, 2007). During somatic embryogenesis, BBM activates auxin biosynthesis *TAA1* and *YUCCA* (*YUC*) genes, ensuring local auxin production which is essential for the maintenance of somatic embryo identity (Fig. 2A) (Horstman *et al.*, 2017b; Karami *et al.*, 2023). In addition, BBM directly activates the *LAFL* transcription factor genes *LEAFY COTYLEDON* (*LEC*)*1* and *LEC2*, *ABSCISIC ACID INSENSITIVE3* (*ABI3*), and *FUSCA3* (*FUS3*) (Horstman *et al.*, 2017b), that act to promote the initiation of somatic embryogenesis and maintenance of embryo identity.

*AGAMOUS-LIKE 15* (*AGL15*) is a target of LEC2 and activates itself and the *LAFL* genes, thereby forming a positive regulatory feedback loop promoting embryogenic development (Braybrook *et al.*, 2006). AGL15 also participates in auxin signaling through the activation of the auxin signaling gene *IAA30* (Fig. 2A) (Wang *et al.*, 2004; Zheng *et al.*, 2016). Remarkably, constitutive *AGL15* expression can maintain a totipotent cell state over years, thereby sustaining somatic embryogenic capacity (Harding *et al.*, 2003). In addition, it was recently found that LEC2 also directly activates *WRINKLED1* (*WRI1*), encoding a key regulator for the biosynthesis of lipids, reserves typically found in seeds. Interestingly, their interaction on the protein level stimulates the induction of LEC2 downstream targets including *LEC1* and *FUS3* (W.J. Zhang *et al.*, 2024). A complex consisting of LEC2, LEC1, FUS3, and WRI1 was shown to activate auxin biosynthesis- and lipid biosynthesis-related genes, suggesting a highly intricate interplay between these two pathways, essential for ensuring successful progression of somatic embryogenesis.

To develop somatic embryos from embryogenic callus, a transfer of the tissue to 2,4-D-free medium is required. Shortly after this transfer, *WUS* is expressed in regions surrounded by high auxin levels. These regions are established by PIN-FORMED (PIN1)-mediated polar auxin transport and resemble the expression domain of *WUS* and auxin accumulation in the shoot apical meristem (Su *et al.*, 2009).

While the diverse modes of regeneration ranging from tissue renewal to somatic embryogenesis involve distinct regulatory mechanisms, they are all promoted by stress and/or hormonal signaling pathways. Most often, multiple intermediate steps are involved in cell fate reprogramming, concomitant with cell cycle progression. Notably, transcriptional activation of stem cell regulators such as *PLT* and *WOX* genes in conjuction with auxin and cytokinin signaling emerge as a common requirement for regeneration.

## Unlocking pluripotency by overcoming epigenetic repression

Chromatin remodeling and epigenetic changes accompany each phase of plant development affecting the expression of genes in cells, tissues, and organs. Not surprisingly, overcoming epigenetic barriers to unlock pluripotency genes is a key aspect of cell fate transition during wound-induced regeneration, *de novo* shoot organogenesis, and somatic embryogenesis (Fig. 1) (Hasegawa *et al.*, 2018; Lee and Seo, 2018; Wang *et al.*, 2020; Wu *et al.*, 2022).

During the various forms of regeneration, phytohormones were found to play a crucial role in regulating the accessibility of genes. For example, the initiation of somatic embryogenesis caused by high auxin concentrations has been shown to lead to a rapid alteration of chromatin accessibility, resulting in activation of cell cycle- and meristem-associated genes (Wang *et al.*, 2020). Here, CHROMOMETHYLASE 3 (CMT3) expression is up-regulated through 2,4-D treatment, and an auxin-responsive motif in its promoter suggests a direct impact on its regulation by ARFs (Grzybkowska *et al.*, 2018). The open chromatin state in combination with expression of the *LAFL* gene seems to be required for somatic embryogenesis (Wang *et al.*, 2020).

Similarly, during induction of *de novo* shoot organogenesis, high auxin promotes the activation of pluripotency-related genes, but the simultaneous presence of cytokinin and its signaling maintains accessibility of shoot identity genes on CIM (Wu *et al.*, 2022). During callus formation, ARF7 and ARF19 recruit the H3K9 demethylase JUMONJI C DOMAIN-CONTAINING PROTEIN30 (JMJ30) to remove the repressive marks from *LBD16* and *LBD19* loci (Lee *et al.*, 2017, 2018), and at the same time ARABIDOPSIS TRITHORAX-RELATED 2 (ATRX2) is recruited by ARFs to deposit the active chromatin mark H3K36me3 to ensure a high *LBD* expression. In addition, the presence of LYSINE-SPECIFIC DEMETHYLASE 1-LIKE 3 (LDL3) erases H3K4me2 marks of downstream genes during callus induction, priming the tissue for later efficient shoot induction (Ishihara *et al.*, 2019). Upon transfer to SIM, the root-associated gene loci rapidly become inaccessible, in contrast to the *WUS* locus where the level of repressive marks is reduced and active transcription marks increase over time (Fig. 1) (Li *et al.*, 2011; Zhang *et al.*, 2017; Wu *et al.*, 2022). ATRX2 is additionally recruited by the type-B ARR1 at the beginning of shoot induction on SIM to activate *ARR5* and *ARR7* expression, which suppresses immediate *WUS* activation (Lee *et al.*, 2021), probably to balance cell fate transition. The different role of ATRX2 in callus or shooting medium indicates that the recruitment of epigenetic modifiers is dependent on the environmental context.

Reprogramming of somatic cells means that marks laid down and maintained in differentiating cells need to be erased. The chromatin modifier POLYCOMB REPRESSIVE COMPLEX (PRC), that is responsible for catalyzing the deposition of repressive H3K27me3 marks, is crucial for maintaining somatic cells in a differentiated state. Specifically, PRC2 is involved in restricting callus and somatic embryo formation by keeping genes such as *WOX5*, *BBM*, and *LAFL* transcriptionally silent (Ikeuchi *et al.*, 2015; Mozgová *et al.*, 2017; Lepiniec *et al.*, 2018; Duarte-Aké *et al.*, 2019). Mutations in subunits of PRC2 result in ectopic growth of vegetative tissue resembling formation of callus or somatic embryos (Cao *et al.*, 2002; Schatlowski *et al.*, 2008; Ikeuchi *et al.*, 2015). The transcriptional repressors VIVIPAROUS1/ABI3-LIKE1 (VAL1) and VAL2 recruit PRC2 to specific genomic regions to control embryogenic development in Arabidopsis (Yuan *et al.*, 2021), and mutations affecting these genes enhance the efficiency of somatic embryogenesis and maintenance of embryo identity (Horstman *et al.*, 2017a; Yuan *et al.*, 2021). Genes targeted by VAL1/2 are also implicated in the wounding response, indicating that the recruitment of PRC2 by VAL1/2 to suppress specific genes plays a crucial role in wound-induced regeneration (Yuan *et al.*, 2021).

In addition to the above, dynamic changes in histone acetylation levels also occur at multiple gene loci during the process of regeneration. For example, genes associated with wound-induced regeneration, such as WIND1, either exhibit pre-existing marks or rapidly accumulate histone acetylation, ensuring a rapid response to injury (Rymen *et al.*, 2019). Experiments with trichostatin A, a histone deacetylase inhibitor, led to spontaneous somatic embryogenesis induction through auxin accumulation and the up-regulation of *LEC1*, *LEC2*, and *BBM* (Wójcikowska *et al.*, 2018). During *de novo* shoot regeneration, the up-regulation of the *HISTONE ACETYLTRANSFERASE1 OF THE GNAT FAMILY 1* (*HAG1*) upon CIM incubation catalyzes the acetylation of histones at loci associated with root stem cell genes including *PLT1/2*, *SCR*, and *WOX5* (Kim *et al.*, 2018). Likewise, HAG1 and HAG3 are required for the up-regulation of *PLT3/5* in mesophyll protoplasts leading to callus induction (Sakamoto *et al.*, 2022). How these (de)acetylation factors are recruited to their targets is not known.

Taken together, auxin and cytokinin signaling create an environment with open chromatin regions at somatic embryo- and stem cell-related genes through the activation of epigenetic modifiers reminiscent of the poised state of developmental genes in animal embryonic stem cells (Azuara *et al.*, 2006; Bernstein *et al.*, 2006; Mikkelsen *et al.*, 2007). Advancing our comprehension of the functional coordination between transcription factors and epigenetic modifiers is crucial for the development of tools aiding genotype-independent regeneration.

## Regeneration by the ectopic expression of developmental regulators

The molecular pathways of the various forms of regeneration culminate in the expression of stem cell-related genes as a requirement to develop a new stem cell niche, a *de novo* shoot, or a somatic embryo. Therefore, to overcome regenerative recalcitrance, the genes that function in stem cells, but also (somatic) embryogenesis and chromatin remodeling, have been strategically employed in conjunction with established hormonal protocols. However, the ectopic expression of developmental regulators comes with a potential drawback of causing pleiotropic effects that negatively influence normal plant development and growth. Here we will provide examples focusing on successful application of developmental regulators while discussing relevant complications as well. We refer the reader to Table 1 for a detailed overview.

**Table 1.** Summary of applied developmental regulatory genes to aid regeneration

Gene cassette	Transformed species	Technique	Hormone	Explant	Regeneration type^*a*^	Regenerated plant	Growth defects	Reference
2 × 35S:HSP189:CRE-CSP:rZmG2	*O. sativa*	Tissue culture	2,4-D, NAA, BAP	Callus	Organogenesis	Yes	No	Luo *et al.* (2023)
35S:GhAGL15-4	*G. hirsutum*	Tissue culture	2,4-D	Leaf petiols	Embryogenesis	NA	NA	Yang *et al.* (2014)
35S:GVG-6×UAS:CcSERK1	*C. canephora*	Tissue culture	NAA, kinetin, BA	Leaf	Embryogenesis	No	No	Pérez-Pascual *et al.* (2018)
PG10_90:XVE-OLexA:AtESR1	*A. thaliana*	Tissue culture	2,4-D, kinetin, IAA, 2-iP	Roots	Organogenesis	Yes	No	Banno *et al.* (2001)
PG10_90:XVE-OLexA:AtESR2	*A. thaliana*	Tissue culture	2,4-D, kinetin, IAA, 2-iP	Flower buds, roots	Organogenesis	Yes	No	Ikeda *et al.* (2006)
35S:AtCUC1/35S:AtCUC2	*A. thaliana*	Tissue culture	2,4-D, IBA, kinetin, IAA	Callus	Organogenesis	Yes	Yes	Daimon *et al.* (2003)
AtMP:AtMPΔ	*A. thaliana*	Tissue culture	2,4-D, IBA, 2-iP	Root, leaf, petiole, cotyledon	Organogenesis	Yes	Yes	Ckurshumova *et al.* (2014)
pER8:PaHAP3A	*P. abies*	Tissue culture	2,4-D, BA, NAA, ABA, IBA	Embryogenic callus	Embryogenesis	No	No	Uddenberg *et al.* (2016)
35S:CsL1L	*C. sinensis*	Tissue culture	2,4-D	Epicotyle, embryogenic callus	Embryogenesis	No	No	Zhu *et al.* (2014)
35S:TcLEC2-GR	*T. cacao*	Tissue culture	2,4-D, TDZ, kinetin	Cotyledon	Embryogenesis	No	No	Shires *et al.* (2017)
PG10_90:XVE-LexA::AtLEC2	*N. tabacum*	Tissue culture	BAP, NAA	Leaf	Organogenesis	Yes	Yes	Rashid *et al.* (2007)
35S:AtAGL15	*A. thaliana*	Tissue culture	2,4-D	Cotyledon	Embryogenesis	No	No	Harding *et al.* (2003)
35S:GmAGL15	*G. max*	Tissue culture	2,4-D	Cotyledon	Embryogenesis	Yes	Yes	Thakare *et al.* (2008)
35S:GhAGL15	*G. hirsutum*	Tissue culture	2,4-D, kinetin, IAA	Hypocotyl	Embryogenesis	No	No	Wang *et al.* (2004)
35S:GVG-UAS:AtRKD4	*A. thaliana*	Tissue culture	Free	Seedling	Embryogenesis	NA	NA	Waki *et al.* (2011)
35S:GAL4-AtRKD4-GR	*Phalaenopsis*	Tissue culture	Free	Leaf	Embryogenesis	Yes	No	Mursyanti *et al.* (2015)
35S:GVG-6×UAS:OsRKD3	*O. sativa* L. cv. Cempo Ireng	Tissue culture	Free	Scutellum	Embryogenesis	Yes	No	Purwestri *et al.* (2023)
35S:ZmKN1	*C. sinensis*	Tissue culture	BAP	Leaf	Organogenesis	Yes	Yes	Wang *et al.* (2004)
35S:ZmKN1	*N. tabacum*	Tissue culture	BAP, NAA	Leaf	Organogenesis	Yes	Yes	Luo *et al.* (2006)
35S:BnSTM; 35S:BoSTM	*B. napus*	Tissue culture	BAP, NAA	Hypocotyl	Organogenesis	NA	NA	Elhiti and Stasolla (2012)
35S:NtNTHs	*N. tabacum*	Tissue culture	BAP, NAA	Leaf	Organogenesis	Yes	Yes	Nishimura *et al.* (2000)
35S:AtGRF5	*C. melo*	Tissue culture	Free	Cotyledons	Organogenesis	Yes	No	Wan *et al.* (2023)
UBI:AtGRF5	*C. lanatus*	Tissue culture	BAP	Cotyledons	Organogenesis	Yes	No	W. Pan *et al.* (2022)
2 × 35S::AtGRF5	*B. vulgaris* ssp. *vulgaris*	Tissue culture	Thidiazuron, GA3	Leaf	Organogenesis	Yes	No	Kong *et al.* (2020)
PcUBI4-2::BnGRF5-LIKE	*B. napus*	Tissue culture	2,4-D, BAP, NAA, GA	Hypocotyl	Organogenesis	Yes	No	Kong *et al.* (2020)
PcUBI4-2::GmGRF5-LIKE	*G. max*	Tissue culture	GA3, kinetin, IAA	Primary node	Organogenesis	Yes	No	Kong *et al.* (2020)
35S::HaGRF5-LIKE	*H. annuus L.*	Tissue culture	NA	Cotyledons	Organogenesis	Yes	Yes	Kong *et al.* (2020)
BdEF1::ZmGRF5-LIKE1	*Z. mays*	Tissue culture	NA	Immature embryos	Organogenesis	Yes	No	Kong *et al.* (2020)
35S:Cl_rGRF4-GIF^*b*^	*C. lanatus*	Tissue culture	BAP, no cytokinin	Cotyledons	Organogenesis	Yes	No	Feng *et al.* (2021)
ZmUBI:wheat_GRF4-GIF1	*T. aestivum*	Tissue culture	Picloram, 2,4D	Immature embryos	Organogenesis	Yes	No	Debernardi *et al.* (2020)
	*O. sativa*	Tissue culture	2,4-D	Embryogenic callus	Organogenesis	Yes	No	
35S:Cl._rGRF4-GIF^*b*^	*C. triptera×C. sinensis*	Tissue culture	BAP, NAA, BAP	Etiolated epicotyls	Organogenesis	Yes	Yes	Debernardi *et al.* (2020)
CsGRF3–CsGIF1	*C. sativa*	Tissue culture	2,4-D, kinetin,BAP, NAA, IAA	Embryo hyopocotyls	Organogenesis	Yes	No	Bull *et al.* (2023)
35S:Grape_rGRF4-GIF1	*Latuca* spp.	Tissue culture	BAP, NAA	Cotyledons	Organogenesis	Yes	No	Bull *et al.* (2023)
35S:PtWOX11	Poplar (*P. alba×P. glandulosa*)	Tissue culture	BA, NAA	Leaf	Organogenesis	Yes	NA	Liu *et al.* (2018)
ZmUBI:TaWOX5	Triticale (*T. aestivum* L. and *T. monococcum*)	Tissue culture	2,4-D, picloram	Immature embryos	Organogenesis	Yes	No	K. Wang *et al*. (2022)
	*Z. mays* L.	Tissue culture	2,4-D, zeatin	Immature embryos	Organogenesis	Yes	No	K. Wang *et al*. (2022)
	*H. vulgare* L	Tissue culture	Dicamba, BAP, kinetin, 1-NAA	Immature embryos	Organogenesis	Yes	No	K. Wang *et al*. (2022)
vspi:MtWUS	*M. tranculata*	Tissue culture	Free	Leaf, roots	Embryogenesis	Yes	No	Kadri *et al.* (2021)
35S:AtWUS	*G. hirsutum*	Tissue culture	2,4-D, kinetin	Hypocotyls	Embryogenesis	Yes	Yes	Bouchabké-Coussa *et al.* (2013)
	*G. hirsutum*	Tissue culture	2,4-D, kinetin, IAA	Hypocotyls	Embryogenesis	No	Yes	Zheng *et al.* (2014)
PG10–90:XVE-OLexA:AtWUS	*C. canephora*	Tissue culture	BAP, IAA	Leaf	Embryogenesis	Yes	Yes	Arroyo-Herrera *et al.* (2008)
	*N. tabaccum*	Tissue culture	BAP, NAA	Leaf, root, hypocotyls	Organogenesis	Yes	Yes	Rashid *et al.* (2007)
35S:MtWOX9_1	*M. tranculata*	Tissue culture	2,4-D, BAP	Leaf	Embryogenesis	Yes	NA	Tvorogova *et al.* (2019)
3×ENH-PLTP:ZmWUS2-HSP26:CRE-NOS:ZmCRC	*Z. mays L.*	Tissue culture	2,4-D, dicamba, ABA, zeatin, BAP	Immature embryos	Embryogenesis	Yes	No	Hoerster *et al.* (2020)
3×ENH-PLTP:ZmWUS2-HSP26:CRE-NOS:ZmCRC	*S. bicolore*	Tissue culture	BAP, ABA, IAA, zeatin	Immature embryos	Embryogenesis	Yes	No	Hoerster *et al.* (2020)
ZmUBI:WOX2a	*Z. mays* L.	Tissue culture	Free	Immature embryos	Embryogenesis	Yes	No	McFarland *et al.* (2023)
PG10–90:XVE OLexA:AtWOX5 or 2/8 or 2/9	*N. tabaccum*	Tissue culture	BAP, NAA	Leaf	Organogenesis	Yes	Yes	Kyo *et al.* (2018)
35S:AtPLT5	*A. majus*	*Agrobacterium* infiltration in excised axillary shoot sides	Free	Axillary meristems	Organogenesis	Yes	Yes	Lian *et al.* (2022)
35S:AtPLT5	*S. lycopersicum*	*Agrobacterium* infiltration in excised axillary shoot sides	Free	Axillary meristems	Organogenesis	Yes	Mild	Lian *et al.* (2022)
35S:AtPLT5	*B. rapa*	Tissue culture	BAP	Root tips	Embryogenesis	Yes	Yes	Lian *et al.* (2022)
35S:AtPLT5	*C. annuum*	Tissue culture	Free	Cotyledons	Embryogenesis	No	NA	
35S:AtPLT5	*C. melo*	Tissue culture	Free	Cotyledons	Organogenesis	Yes	No	Wan *et al.* (2023)
35S:MdAIL5	*M. domestica*	Tissue culture	BAP, IAA, GA3	Leaf, shoot	Organogenesis	Yes	No	K. Liu *et al.* (2023)
35S:AtBBM	*A. thaliana*, *B. napus*	Tissue culture	BAP	Leaf, hypocotyl	Embryogenesis	Yes	Yes	Boutilier *et al.* (2002)
35S:AtBBM	*T. cacao*	Tissue culture	Free	Leaf, shoot	Organogenesis	No	NA	Florez *et al.* (2015)
35S:AtBBM	*N. tabaccum*	Tissue culture	IAA	Hypocotyl	Embryogenesis	Yes	Yes	Srinivasan *et al.* (2007)
35S:BnBBM;35S:BnBBM-GR	*N. tabaccum*	Tissue culture	Zeatin, BAP	Hypocotyl	Embryogenesis	Yes	Yes	Srinivasan *et al.* (2007)
ZmUBI::OsBBM-GR	*O. sativa*	Tissue culture	Free	Seeds	Embryogenesis	No	NA	Khanday *et al.* 2023
35S:TcBBM	*T. cacao*	Tissue culture	Free	Cotyledons, floral buds	Embryogenesis	No	NA	Florez *et al.* (2015)
dCas9-sgRNA(BBM)SunTag	*O. sativa*	Tissue culture	Free	Callus	Organogenesis	Yes	No	C. Pan *et al*. (2022)
HSP17:CRE-Nos:ZmWUS2-3×ENH -Ubi:ZmBBM	*Z. mays*, *S. bicolor, P. virgatum*, *C. americanus*, *S. italica*, *S.cereale*, *H. vulgare*, *O. sativa*	Tissue culture	2,4-D, ABA, BAP, IAA, zeatin, IBA	Leaf	Embryogenesis	Yes	No	N. Wang *et al.* (2023)
RAB17:CRE-Nos:ZmWUS2-Ubi:ZmBBM	*Z. mays*, *O. sativa*, *S. bicolor*, *Saccharum officicarium*	Tissue culture	2,4-D, BAP	Immature embryo, mature seed, leaf, callus	Embryogenesis	Yes	No	Lowe *et al.* (2016)
ZmPLTP:ZmBBM-ZmAxig1:ZmWUS2	*Z. mays*	Tissue culture	2,4-D, BAP	Immature embryos	Embryogenesis	Yes	No	Lowe *et al.* (2018)
HSP2:CRE-ZmPLTP:ZmBBM-ZmPLTP:ZmWUS2	*S. bicolor*	Tissue culture	Free	Immature embryos	Embryogenesis	Yes	No	Aregawi *et al.* (2022)
PLTP:ZmBBM_ZmUBI:TaGRF4-GIF1	*Z. mays*	Tissue culture	2,4-D, dicamba, BAP	Immature embryos	Embryogenesis	Yes	No	Chen *et al.* (2022, Preprint)
Nos:WUS2-AtUBI:STM	*N. benthamiana*	Fast-TrACC^*c*^	Free	Seedlings	Organogenesis	Yes	Yes	Maher *et al.* (2020); Cody *et al.* (2023)
	*N. benthamiana*	*Agrobacterium* infiltration in excised axillary shoot sides	Free	Mature plants	Organogenesis	Yes	Yes	
Nos:ZmWUS2; 35S:ipt; 35S:STM; 35S:MT△; AtUBi:BBM^*d*^	*S. lycopersicum*	Fast-TrACC^*c*^	Free	Seedlings	Organogenesis	Yes	NA	Maher *et al.* (2020)
	*S. tuberosum*, *V. vinifera*	*Agrobacterium* infiltration in excised axillary shoot sides	Free	Mature plants	Organogenesis	Yes	Yes	Maher *et al.* (2020)
PG10–90:GVG-UAS:PLT1-UAS:WOX5	*A. thaliana*, *S. lycopersicum*	Tissue culture	Free	Seedlings	Organogenesis	Yes	No	Scheres *et al.* (2019)

^*a*^ miR396-resistant GRF4.

^*b*^ Embryogenesis, somatic embryogenesis.

^*c*^ Fast-treated *Agrobacterium* co-culture.

^*d*^ Separate vectors simultaneously injected.

NA, not applicable.

Consistent with the cytokinin-sensitizing role of WOX5, its overexpression during callus induction was shown to enhance the shoot regeneration capacity in various species (Lee *et al.*, 2022; K. Wang *et al*., 2022; Zhai *et al.*, 2023). Striking is the direct shoot induction by *WOX5* or *WUS* overexpression in the absence of hormones, although this occurred exclusively from root meristem tissue (Gallois *et al.*, 2004; Rashid *et al.*, 2007; Rashid and Kyo, 2009). Similarly, artificially elevated cytokinin levels allowed the conversion of stem cells from developing lateral root primordia into shoot stem cells, promoting subsequent shoot outgrowth in the absence of callus formation (Rosspopoff *et al.*, 2017). These results confirm the ‘poised’ properties of root meristematic cells to follow a root or shoot trajectory depending on the input.

In agreement with the requirement of *AIL*/*PLT* genes for shoot regeneration, the effectiveness of ectopic *PLT5* expression in improving shoot regeneration and in overcoming recalcitrance has been demonstrated across various plants species such as snapdragon, tomato, melon, and cabbage (Lian *et al.*, 2017; Wan *et al.*, 2023). Downstream up-regulation of *PLT1/2* to establish pluripotency, and activation of *CUC* gene expression preceding shoot primordia formation was confirmed in the callus stage (Kareem *et al.*, 2015; Lian *et al.*, 2017). *PLT5*, similar to *BBM* overexpression, also induced somatic embryos, and these could convert to independent plants without the need for exogenous hormones (Boutilier *et al.*, 2002; Tsuwamoto *et al.*, 2010). However, overexpression of *BBM* in rice and *Theobroma cacao* initiated somatic embryogenesis but did not result in plant outgrowth (Florez *et al.*, 2015; Khanday *et al.*, 2023; Alburqueque-Vasquez *et al.*, 2023), demonstrating that *AIL/PLT* expression alone does not suffice for plant regeneration in all species.

The ectopic expression of BBM downstream targets can also promote the transition from vegetative to embryonic identity and does so in various species. In Arabidopsis, this transition is induced by *LEC1*, *LEC2*, and *AGL15*, and in soybean and cotton by ectopic *AGL15* (Lotan *et al.*, 1998; Thakare *et al.*, 2008; Yang *et al.*, 2014). Ectopic *LEC2* improved somatic embryo formation in economically important but regeneration-recalcitrant species such as cassava and *T. cacao*, which correlated with an up-regulation of *FUS3* and *ABI3*, as well as increased auxin levels (Shires *et al.*, 2017; Brand *et al.*, 2019). Notably, whereas in angiosperms *LEC1* could induce embryonic properties from vegetative tissue (Guo *et al.*, 2013; Zhu *et al.*, 2014), in conifers, such as *Picea abies*, somatic embryos could only be induced from embryonic tissue (Uddenberg *et al.*, 2016).

Recently, transcription factors related to the earliest embryo developmental stages such as WOX2 and RWP-RK DOMAIN-CONTAINING PROTEIN (RKD) were found to be useful in the improvement of regeneration. Overexpression of *RKD4* in Arabidopsis induced somatic embryo formation (Waki *et al.*, 2011) whereas overexpression of a rice homolog *OsRKD3* was even able to break recalcitrance of black rice by activating the embryonic program and repressing vegetative development (Purwestri *et al.*, 2023). *WOX2* was identified to play a crucial role in somatic embryogenesis by exploiting natural variation of maize inbred lines. Subsequent overexpression of *WOX2* improved somatic embryo formation in recalcitrant inbred lines. Importantly, *WOX2* overexpression lines did not display aberrant phenotypes, which is beneficial for its application, but also suggests the involvement of additional factors during the reprogramming at the immature embryo explant stage (McFarland *et al.*, 2023).

Finally, an important observation is that overexpression of *WUS* not only results in shoot organogenesis but also facilitated the transition from vegetative to embryogenic fate (Zuo *et al.*, 2002; Gallois *et al.*, 2004; Xiao *et al.*, 2018). The dual functionality of WUS is believed to be correlated with auxin levels, with low concentrations inducing shoot organogenesis and high concentrations leading to somatic embryogenesis (Gallois *et al.*, 2004; Xiao *et al.*, 2018). Indeed, it was shown that constitutive or induced overexpression of *WUS* in the presence of auxin enhanced somatic embryogenesis significantly in many crop species (Rashid *et al.*, 2007; Arroyo-Herrera *et al.*, 2008; Solís-Ramos *et al.*, 2009; Zheng *et al.*, 2014; Lowe *et al.*, 2018; Hoerster *et al.*, 2020; Kadri *et al.*, 2021; Che *et al.*, 2022). However, the overexpression of AtWUS in the conifer *Picea glauca* and in *Capsicum chinense* could promote embryogenic structures but did not result in the development of somatic embryos (Solís-Ramos *et al.*, 2009; Klimaszewska *et al.*, 2010), suggesting again that other signals are required here to complete the embryogenic development.

Together, the widespread impact of WUS points to a conserved role in orchestrating pluri- and totipotency within the plant kingdom. Besides *WUS*, the above-mentioned *PLT5* and *LEC2* also display a dual functionality towards *de novo* shoot organogenesis and somatic embryogenesis which may rely predominantly on the inductive conditions. Together, this argues against the strict separation of shoot organogenesis and somatic embryogenesis processes. Both are the result of self-organization, the origin of which is determined by the inductive cues highlighting the plasticity of plant cells.

In addition, the above examples indicate that additional factors, for example related to the hormonal balance, cellular status of a tissue, or species specific, can be decisive in augmenting the effect of the ectopic developmental regulator. Studies towards the identification of these factors will enhance our understanding on regeneration as well as provide additional tools to break recalcitrance.

### The synergistic effects of multiple developmental regulators enhance regeneration

To successfully reprogram somatic cells into iPSCs in mammals, the OSKM factors need to be expressed together. Several studies have advocated the concurrent use of multiple (transcription) factors also in plants, providing synergistic effects compared with application of either factor alone.

In a groundbreaking study, the combination of *ZmWUS2* and *ZmBBM* was used to effectively boost regeneration efficiency in numerous recalcitrant maize inbred lines (Lowe *et al.*, 2016). Subsequently, this combination was found to enhance regeneration in various other crop species such as *Sorghum bicolor*, rice, *Saccharum officinarum*, *Panicum virgatum*, and *Eragostis tef*, and thereby lifted the transformation constraints for many monocot crops (Lowe *et al.*, 2016; Beyene *et al.*, 2022; Xu *et al.*, 2022; J. Wang *et al*., 2023).

Combining the shoot meristem factor gene *ZmWUS* with *AtSTM* or the bacterial *IPT* cytokinin biosynthesis gene facilitated organogenesis of dicot species such as Arabidopsis, *Nicotiana benthamiana*, tomato, potato, and grape (Maher *et al.*, 2020). Also in Arabidopsis, the expression of *miR964* together with *WUS* increased somatic embryogenesis competence of the recalcitrant ecotype Landsberg *erecta* (Lu *et al.*, 2023), probably by potentiating the effect of WUS in a manner reminiscent of its role in the shoot apical meristem. The fact that *miR964* appears to be evolutionarily ancient and its function is conserved within the plant kingdom (Kumar *et al.*, 2019) warrants testing of its combined overexpression with *WUS* in also overcoming recalcitrance in crop species.

Chromatin remodeling is a key event during the reprogramming events leading to regeneration, and by directly influencing the chromatin state can speed up and aid in regeneration efficiency. Very effective are the GROWTH REGULATORY FACTORs (GRFs) that form a complex with their transcriptional cofactors GRF INTERACTING FACTOR (GIF) to regulate target genes in a variety of plant developmental processes (reviewed in Liebsch and Palatnik, 2020). During callus formation, the GRF–GIF complex is thought to recruit SWITCH/SUCROSE NONFERMENTING (SWI/SNF) chromatin-remodeling complexes to promote meristematic identity, facilitate organogenesis, and promote proliferation. Accordingly, GRFs have been shown to play a pivotal role in enhancing the regeneration process across diverse recalcitrant crops, such as different melon species, red beet, canola, soybean, and sunflower, and promoting embryogenesis in maize (Kong *et al.*, 2020; W. Pan *et al.*, 2022). Chimeric fusions of the GRF and GIF1 coding sequences demonstrated even greater regeneration efficiency (Debernardi *et al.*, 2020; Feng *et al.*, 2021; Zhang *et al.*, 2021; Bull *et al.*, 2023). In addition, mutating the *miRNA396-*binding site that post-transcriptionally regulates some *GRF* family members (Liebsch and Palatnik, 2020; X. Liu *et al.*, 2023) has emerged as a successful strategy in achieving elevated GRF protein levels and enhancing regeneration in specific crops such as watermelon, citrus, and lettuce (Kong *et al.*, 2020; Feng *et al.*, 2021; Bull *et al.*, 2023).

The combination of *ZmBBM* and *TaGRF4-GIF1* enhanced the regeneration of transformed maize seedlings 7-fold compared with the isolated use of either gene (Chen *et al.*, 2022, Preprint).

However, most of the regeneration studies utilizing a combination of developmental regulators in grass species still rely on the use of immature embryos, practically limiting the success to only a few major *Poaceae* crops. Optimizing activity of the promoter driving the developmental regulators facilitated the regeneration of transgenic plants from leaf blades in various *Poaceae* species (N. Wang *et al*., 2023), indicating the importance of dosage.

### The significance of timing and dosage in the application of developmental regulators

Regeneration is a multifaceted process that unfolds through distinct phases, governed by genes driving dedifferentiation, pluripotency/totipotency acquisition, and shoot/plant formation (Fehér, 2019; Shin *et al.*, 2020). Constitutive overexpression of developmental regulators can facilitate the regeneration process as discussed above, yet frequently results in unfavored pleiotropic effects in the transgenic progeny (Boutilier *et al.*, 2002; Daimon *et al.*, 2003; Wang *et al.*, 2004; Hu *et al.*, 2005; Luo *et al.*, 2006; Rashid *et al.*, 2007; Srinivasan *et al.*, 2007; Lian *et al.*, 2017; Kyo *et al.*, 2018). Employing timely or sequential gene induction specific to each stage of regeneration could be crucial for effective embryo or organ development. For example, in the presence of constitutive *miR394* overexpression, the induction of *WUS* before the embryogenic callus formation step most effectively enhanced somatic embryo formation, compared with its induction at later time points (Lu *et al.*, 2023).

To control when and where the developmental regulators are to be activated, several strategies have been developed such as excision systems, tissue-specific promoters, or the use of inducible systems (Moore *et al.*, 2006; Borghi, 2010; Ali and Kim, 2019; Ochoa-Fernandez *et al.*, 2020; Maren *et al.*, 2022). A combination of chemically inducible systems was used to enhance regenerative abilities of somatic cells by sequentially inducing *WIND1* and *LEC2* transcription factors in Arabidopsis (Iwase *et al.*, 2015). Using a dexamethasone-inducible system, the activation of LEC2 alone, fused with the hormone-binding domain of the rat glucocorticoid receptor (GR), resulted in few embryogenic callus loci in etiolated hypocotyls. However, the sequential expression of *WIND1*, via an estradiol-inducible transactivation system, followed by LEC2–GR activation substantially increased the number of regeneration-competent cells and thereby somatic embryo formation, without requiring added phytohormones. This study suggests that WIND1 has the potential to enhance developmental plasticity and, when combined with other developmental regulators, facilitates the reprogramming of plant cell fate to acquire new characteristics. The uncoupling of different regeneration stages would also allow for an in-depth study into each phase, including the mechanisms behind pluri- and totipotency acquisition.

With the increasing CRISPR/Cas [clustered regularly interspaced palindromic repeats (CRISPR)/CRISPR-associated protein] toolkit, there appears to be no limit to the number of genes that can be activated via single guide RNA (sgRNA) targeting, meaning that the practical construct size limitations fade. By applying an inducible SunTag synthetic transcription activator, here containing a deadCas9 that is targeted to activate several developmental regulator gene loci by co-expressed sgRNAs, improved transformation, and regeneration was obtained in strawberry and sheepgrass (C. Zhang *et al*., 2024). Combined with an additional induction system, such a set-up can interrogate the effect of combinations of genes not only simultaneously but also sequentially.

In addition to timing, the dosage of gene activation may be regulated based on the concentration of inducer molecules. The significance of dosage was highlighted by experiments demonstrating that low expression of *WUS* in combination with strong expression of *BBM* significantly enhanced somatic embryogenesis in monocot species, compared with driving both genes from strong promoters (Lowe *et al.*, 2016). In agreement, excessively high induction of a WUS–GR fusion proved detrimental to somatic embryogenesis induction in Arabidopsis (Lu *et al.*, 2023).

In the context of animal iPSCs, various DNA-free delivery methods have been developed over the years to overcome integrative approaches, thereby enhancing the clinical applicability of iPSCs in humans (Borgohain *et al.*, 2019). To alleviate regulatory constraints and public opposition towards the production of genetically engineered corps, transient expression, or protein delivery into various plant tissues, protoplasts, or cell suspensions are enjoying increased research attention (Kocsisova and Coneva, 2023; Nagy *et al.*, 2023; Nakamura *et al.*, 2023). The use of rhabdovirus to transiently deliver expression cassettes (Ma *et al.*, 2020) as well as protein delivery via nanoparticles (Nagy *et al.*, 2023) are options in plants. Direct delivery of proteins into cells theoretically allows for control over dosage and timing of application, and thereby enables the administration of sequential stimulations with different developmental regulators. Recently, cell-penetrating peptides (CPPs) were used to deliver WUS protein into Arabidopsis seedlings, where they could move from the cytosol into the nucleus and recapitulate the WUS-specific transcriptional downstream response. However, WUS-mediated somatic embryogenesis was not observed (Nagy *et al.*, 2023), suggesting that different levels of WUS protein or additional factors are required. In light of this, the effects of the repressive WOX13 during shoot induction demonstrate that attention should also be paid to the inhibitory elements in the regeneration process. Their mutation may sensitize tissues for pluripotency or totipotency acquisition during reprogramming, thereby aiding in the effect of developmental regulators. Indeed, the repression of vegetative development in favor of regenerative capacity is an essential part of reprogramming, as observed for the mutation of the *bHLH041* repressor gene that favored callus formation and subsequent shoot regeneration (Xu *et al.*, 2023). Similarly, the regenerative potential of different maize inbred lines could be linked to single nucleotide polymorphisms (SNPs) in the promoter region of the maize *SMALL AUXIN UPREGULATED RNA15* (*ZmSAUR15*). ZmSAUR15 inhibits embryogenic callus induction, presumably by blocking the biosynthesis of auxin in callus tissue. Subsequent knockout mutations of *ZmSAUR15* strongly improved embryogenic callus formation in maize (Y. Wang *et al*., 2022).

Together these results make a case for the importance of timing and levels in terms of both developmental regulator application and sensitizing tissues for response.

## Perspective: navigating plant regeneration beyond phytohormones

Following the iPS concept in mammals, plant regulatory genes associated with stem cell regulation and embryo development are now being used to promote pluri- and totipotency, thereby enhancing regeneration processes while holding on to the already developed hormone-dependent protocols. Another interesting approach was recently described to improve plant regeneration efficiency through the application of small peptides. By applying small peptides derived from the WUS sequence that are conserved between family members to callus tissue from *Eucalyptus*, an increased regeneration potential was observed, thereby circumventing the need to overexpress the gene. The peptide treatment activated carbohydrate metabolic pathways including those of starch and sucrose, which play an essential part in callus and shoot formation (Lee and Huang, 2014; Omary *et al.*, 2023; Zhang *et al.*, 2023). Similarly, the plant elicitor peptide REGENERATION FACTOR 1 (REF1), a system-independent local wounding signal, promotes the activation of *WIND1* when applied during tissue culture and enhanced regeneration in many recalcitrant crops, including both monocts and dicots (Yang *et al.*, 2024).

Nevertheless, an ideal situation would be to apply a standard set of developmental regulators that, independent of hormone addition, could induce regeneration in a truly species- and genotype-independent way. Hormone-independent induction of regeneration may also provide a more controlled and clear insight into the intrinsic genetic framework underlying the reprogramming of somatic cells to the pluripotent or totipotent state. Even though our knowledge on the molecular function of master transcription factors and their cofactors is advancing, predicting to what target loci they will bind and what the transcriptional output might be in a specific cell type and stage remains imprecise. Therefore, we propose systematic approaches to test known regulators involved in regeneration and stem cell niche specification as was initiated by Takahashi and Yamanaka in the animal field. A start was made towards this end in the successful application of root stem cell regulator combinations to accomplish hormone-free regeneration in Arabidopsis and tomato (Scheres *et al.*, 2019).

Unlike the situation in animals, where iPSCs can be cultured *in vitro* using appropriate growth factors, the challenge in plants persists in maintaining pluripotent stem cells outside of their native stem cell niches. Although speculative, pluripotent or even totipotent plant stem cell cultures would hold great potential for research as well as for application. However, our ability to establish stem cell cultures requires insight into the transcriptional profile of a totipotent or pluripotent cell, the cues needed to keep this status, and the signals that stem cells need in order to adopt a specific differentiated fate.

In conclusion, direct reprogramming of somatic cells has been proven to be an extraordinarily tractable and insightful tool in the animal field to systematically dissect developmental potential, differentiation, and cellular states. Applying the advances in molecular genetics and single-cell technologies, it will be now possible to study stem cell characteristics and cell state transitions more deeply in plants. We envisage that such an endeavor will help to answer the outstanding question: How can we effectively reconstitute and maintain a plant stem cell niche for regenerative purposes, with the goal of creating a genotype-independent tool for tissue regeneration? Already the positively acting peptides and negative regulator mutants can be directly applied. The challenge then is to translate the future results gained from developmental regulator-based approaches, often being performed in model species, into practical applications for crops.
